# Modulation of Glucose Metabolism by Balanced Deep-Sea Water Ameliorates Hyperglycemia and Pancreatic Function in Streptozotocin-Induced Diabetic Mice

**DOI:** 10.1371/journal.pone.0102095

**Published:** 2014-07-11

**Authors:** Byung Geun Ha, Jung-Eun Park, Eun Ji Shin, Yun Hee Shon

**Affiliations:** Bio-Medical Research Institute, Kyungpook National University Hospital, Daegu, Korea; Consiglio Nazionale delle Ricerche, Italy

## Abstract

The aim of this study was to determine the effects of balanced deep-sea water (BDSW) on hyperglycemia and glucose intolerance in streptozotocin (STZ)-induced diabetic mice. BDSW was prepared by mixing DSW mineral extracts and desalinated water to yield a final hardness of 1000–4000 ppm. Male ICR mice were assigned to 6 groups; mice in each group were given tap water (normal and STZ diabetic groups) or STZ with BDSW of varying hardness (0, 1000, 2000, and 4000 ppm) for 4 weeks. The STZ with BDSW group exhibited lowered fasting plasma glucose levels than the STZ-induced diabetic group. Oral glucose tolerance tests showed that BDSW improves impaired glucose tolerance in STZ-induced diabetic mice. Histopathological evaluation of the pancreas showed that BDSW restores the morphology of the pancreatic islets of Langerhans and increases the secretion of insulin in STZ-induced diabetic mice. Quantitative real-time PCR assay revealed that the expression of hepatic genes involved in gluconeogenesis, glucose oxidation, and glycogenolysis was suppressed, while the expression of the genes involved in glucose uptake, β-oxidation, and glucose oxidation in muscle were increased in the STZ with BDSW group. BDSW stimulated PI3-K, AMPK, and mTOR pathway-mediated glucose uptake in C_2_C_12_ myotubes. BDSW increased AMPK phosphorylation in C_2_C_12_ myotubes and improved impaired AMPK phosphorylation in the muscles of STZ-induced diabetic mice. Taken together, these results suggest that BDSW is a potential anti-diabetic agent, owing to its ability to suppress hyperglycemia and improve glucose intolerance by modulating glucose metabolism, recovering pancreatic islets of Langerhans and increasing glucose uptake.

## Introduction

In recent years, the number of diabetes patients has steadily increased worldwide. The pathologic mechanisms of this disease are mainly attributed to impaired insulin secretion by pancreatic β-cells and insulin resistance in target tissues, including skeletal muscle and the liver, which ultimately leads to hyperglycemia. A potential cause of diabetes may be dysfunctional insulin-secreting pancreatic β-cells that do not have the capacity to secrete insulin; additionally, glucose metabolism dysfunction, specifically glucose production and glucose uptake, may play a role in this disease [1]. Currently, the available diabetic therapies include insulin and several oral anti-diabetic agents such as sulfonylureas, thiazolidinediones, and α-glucosidase inhibitors; these drugs can be used as a monotherapy or in combination, to achieve optimal glycemic control. However, studies of both basic sciences and clinical trials indicate that sulfonylureas cause hypoglycemic symptoms associated with persistent insulin secretion. These effects are not dependent on blood glucose levels and attenuate the efficacy of long-term diabetes treatments. In addition, most oral anti-diabetic agents are associated with many serious adverse effects [2]. Therefore, the development of novel anti-diabetic agents that affect various physiological targets is critical.

It is well known that diabetes, gastrointestinal disorders, and other disorders are related to significant mineral deficiencies and imbalances [3]. Perturbations to the metabolism of trace minerals such as chromium (Cr), selenium (Se), iron (Fe), zinc (Zn), copper (Cu), and manganese (Mn) also can be induced by these diseases and their complications [4]. The link between diabetes mellitus and magnesium (Mg) deficiency is particularly well studied. Emerging evidence suggests Mg plays a pivotal role in reducing the risk of cardiovascular disease and may be involved in the pathogenesis of diabetes itself [5]. Although the benefits of oral Mg supplementation on glycemic control have yet to be demonstrated in diabetes patients, Mg supplementation has been shown to improve insulin sensitivity. Many studies show that mean plasma levels are lower in patients with both type 1 and type 2 diabetes than in non-diabetic controls. In individuals with diabetes and insulin resistance, the intracellular concentration of free Mg in erythrocytes is a more sensitive marker than plasma levels of Mg. Most patients with type 2 diabetes have decreased levels of free intracellular Mg in erythrocytes [6] [7].

Deep-sea water (DSW) is a safe, stable natural resource and is available in infinite supply as compared to other natural products. It contains high levels of unique minerals such as magnesium (Mg), calcium (Ca), and potassium (K), as well as important trace minerals such as Cr, Se, Zn, and vanadium (V). Several studies investigating the physiological consequences of DSW at various degrees of hardness found that DSW might aid in preventing hypertension [8], atopic eczema/dermatitis syndrome [9], and arteriosclerosis [10]. Our recent study also showed that balanced DSW (BDSW) has anti-diabetic potential [11] by suppressing hyperglycemia and improving glucose intolerance by increasing glucose uptake. In addition, BDSW has anti-obesity potential [12] by inhibiting adipocyte hypertrophy and reducing liver steatosis in high-fat diet-induced obese diabetic mice.

On the basis of the abovementioned scientific evidence, we hypothesized that BDSW with high beneficial mineral content influences the development and progression of diabetes. In present study, to get a more profound estimation of the potential of BDSW on development of diabetes, we investigated the effects of BDSW on blood glucose levels, glucose tolerance, pancreatic function, and glucose uptake in STZ-induced diabetic mice.

## Materials and Methods

### Materials

C_2_C_12_ cells were purchased from the American Type Culture Collection (ATCC No.CRL-1772, Manassas, VA, USA). Male ICR mice (4 weeks of age) were obtained from Charles River Japan (Charles River Japan, Kanagawa, Japan). The following items were purchased from the cited commercial sources: 5-aminoimidazole-4-carboxamide 1-β-D-ribofuranoside, acadesine, N1-(β-D-ribofuranosyl)-5-aminoimidazole-4-carboxamide (AICAR) and 3, 3-diaminobenzidine tetrahydrochloride (DAB) from Sigma-Aldrich Co. LLC. (St. Louis, MO, USA); Glucose, Cleantech TG-S, and ASAN SET Total-Cholesterol assay kit from Asan Pharmaceutical Co. Ltd. (Seoul, Korea); Ultra Sensitive Mouse Insulin ELISA kit from Crystal Chem Inc. (Downers Grove, IL, USA); Mouse Adiponectin/Acrp30 and Leptin ELISA kit from R&D Systems, Inc. (Minneapolis, MN, USA); Mouse IL-6 and TNF-α ELISA kit from Biolegend, Inc. (San Diego, CA, USA); anti-phospho IRS1, anti-phospho LKB1, anti-phospho mTOR, and anti-phospho AMPK from Cell Signaling Technology (Danvers, MA, USA); guinea pig anti-human insulin, and rabbit anti-glucagon from Merk Millipore Co. (Billerica, MA, USA); peroxidase-conjugated AffiniPure goat anti-rabbit Ig (H+L) from proteintech group Inc. (Chicago, IL, USA); anti-β-actin, horseradish peroxidase (HRP)-conjugated anti-mouse IgG, and anti-rabbit IgG-HRP antibodies from Santa Cruz Biotechnology Inc. (Santa Cruz, CA, USA); ECL Plus Western Blotting Substrate from Pierce Biotechnology (Rockford, IL, USA); trizol and 2-(N-(7-nitrobenz-2-oxa-1,3-diazol-4-yl)amino)-2-deoxyglucose (2-NBDG) from Invitrogen Life Technologies (Carlsbad, CA, USA); PrimeScript 1st strand cDNA Synthesis Kit from Takara Bio Inc. (Shiga, Japan); FastStart Universal SYBR Green Master from Roche Applied Science (Basel, Switzerland); Phosphatase Inhibitor Cocktail and Protease Inhibitor Cocktail solutions from GenDEPOT (Barker, TX, USA).

### The preparation of BDSW

Original DSW that had been pumped up from a depth of 0.5 km and a distance of 6.7 km off Oho-Ri, Goseong (Gangwon-Do, Korea) was filtered using a microfilter system (Synopex INC, Pohang, Korea) to remove phytoplankton and marine microorganisms. The filtered DSW was passed through a reverse osmosis membrane (Vontron Technology Co., Ltd., Beijing, China), and DSW mineral extracts and desalinated water were obtained. Next, the DSW mineral extracts and the desalinated water were mixed to prepare BDSW having an Mg:Ca ratio of 3∶1. The BDSW was serially diluted with regard to hardness. BDSW used in this study did not include any peptides or organic molecules. In this study, we defined the hardness of BDSW by focusing on the concentrations of Mg and Ca. The hardness values were calculated according to the following equation: Hardness  = Mg (mg/L) ×4.1+ Ca (mg/L) ×2.5. The mineral content of samples were measured by Dionex ICS-1100 basic integrated ion chromatography system (Thermo Scientific Inc., Sunnyvale, CA, USA) for anion analysis such as Ca, Mg and ELAN 9000/6X00/DRC-e ICP-MS (PerkinElmer Inc., Waltham, MA,USA) for trace minerals analysis such as Se, V, and Zn. Table S1 shows the mineral content of the original and BDSW samples with a hardness of 4680 ppm. All of the BDSW used in this study was sterilized by passing it through a 0.2- µm bottle-top filter (Fisher Scientific Inc., IL, USA).

### Animals and induction of diabetes

All animal experiments were conducted in accordance with the guidelines established by the Animal Ethics Committee of Kyungpook National University, and the protocols were approved by this committee (Approval No. KNU 2012–88). To determine the effect of BDSW on fasting blood glucose levels, ICR mice were used as the animal model. All of the experiments in this study were conducted on 4–9-week-old male littermates. To induce diabetes condition, diabetes was induced in overnight fasted ICR mice by intraperitoneal administration with STZ (50 mg/kg body weight), dissolved in 0.1 M citrate buffer (pH 4.5). Mice injected with an equal volume of 0.1 M citrate buffer were maintained as normal groups. The mice were housed in an air-conditioned room with a temperature of 22±2°C, a relative humidity of 40±5% and an 8:00–20:00 light cycle. All the mice were maintained on a stock CRF-1 pellet diet (Oriental Yeast Co., Tokyo, Japan). The more detailed information about standard ingredients of CRF-1 were listed in Table S2. After preliminary feeding for 1 week, the ICR mice (4 weeks of age) were divided into 6 groups (N = 10 per group) with similar fasting blood glucose levels and body weights. The ICR mice in each of the 6 groups were given either the tap water (normal group and STZ diabetic control group) or STZ with BDSW of different hardness (0, 1000, 2000, and 4000 ppm) for 4 weeks. The weight and intake of water and food were measured every 2 days. Water and food were always available and changed every 2 days. After the mice were deprived of their diet for 4 h, blood was collected every week to determine fasting blood glucose levels as described below. At the end of the feeding period, blood was collected from the tail vein, followed by exsanguination under anesthesia with zoletil (Virbac S.A, Carros, France).

### Determination of blood glucose level

The effect of BDSW on fasting blood glucose levels was examined using a previously described procedure [11]. Briefly, mice were deprived of their diet until blood collection from the tail vein 4 h later but allowed free access to water. Blood (10 µl) was added to water (30 µl), 20% (w/v) trichloroacetic acid aqueous solution (40 µl) was added, and test tubes containing the mixture were kept in ice-cold water. The mixture was then centrifuged at 12000×g and 4°C for 10 min. The resultant supernatant (10 µl) was subjected to glucose determination using the Glucose Test Kit (Asan Pharmaceutical Co. Ltd.) and the absorbance at 505 nm was measured using a spectrophotometer (VersaMax microplate reader; Sunnyvale, CA, USA).

### Oral glucose tolerance test (OGTT)

The OGTT was performed at 4 weeks after BDSW feeding. Briefly, a normal group, a diabetic control group, and diabetic 4 groups with BDSW (hardness, 0, 1000, 2000, and 4000 ppm) were deprived of their diet but allowed free access to water. After fasting for 16 h, blood was collected from the tail veins of all mice (0 min). Immediately after blood collection, all mice received an intraperitoneal injection of glucose (0.2 g/100 g body weight). Blood samples were successively collected at the indicated time intervals (0, 30, 60, 90, and 120 min), and blood glucose levels were determined as mentioned above.

### Triglyceride and cholesterol assays of the liver and serum

Serum triglyceride and total cholesterol concentrations were measured enzymatically using commercially available kits (Cleantech TG-S and ASAN SET total-cholesterol assay kit) according to the manufacturer's instructions. Hepatic lipids were extracted as described previously with some modifications [13]. For the cholesterol and triglyceride assays, the dried lipid residues were dissolved in 1 ml ethanol. Hepatic cholesterol and triglycerides were analyzed using the same enzymatic kit as that used in the plasma analysis.

### Histopathological analysis of pancreas

Pancreas tissues were fixed in 4% formalin over 24 h. Fixed tissues were processed routinely for paraffin embedding. Insulin and glucagon were detected on the 5- µm pancreatic sections by guinea pig anti-human insulin diluted 1/100 (Millipore Corp., Billerica, MA, USA) and rabbit anti-glucagon diluted 1/3,000 (Millipore Corp., Billerica, MA, USA), respectively, followed by incubation with peroxidase-conjugated AffiniPure goat anti-rabbit Ig (H+L) diluted 1/100 (Proteintech Group Inc., Chicago, USA). 3, 3-diaminobenzidine tetrahydrochloride (DAB: Sigma-Aldrich, St. Louis, MO, USA) was applied to the sections as the substrate for peroxidase. The sections were counterstained by hematoxylin. Image acquisition was performed using an optical microscope (Nikon eclipse 80i; Tokyo, Japan) with a magnifying power of 400.

### Cell culture and myotube differentiation

C_2_C_12_ cells were maintained in DMEM containing 10% FBS and 1% antibiotics (penicillin-streptomycin) in 5% CO^2^ at 37°C. To induce differentiation, 2 d post-confluent C_2_C_12_ cells were incubated in differentiation induction medium containing DMEM with 2% horse serum. The media were changed daily, and myotubes were ready for experiments 4–5 days after plating.

### Determination of glucose uptake in C_2_C_12_ myotubes

Glucose uptake in C_2_C_12_ myotubes was measured using 2-NDBG as previously described [14]. Mature C_2_C_12_ myotubes differentiated on 96-well black culture plates (Greiner Bio-One, CELL STAR 96W Plate, NC, USA) were washed with PBS. The cells were starved with serum-free low-glucose DMEM for 1 h before incubating with DMEM containing different levels of BDSW hardness and 20 µM 2-NBDG in the presence or absence of 1 mM AICAR and 100 nM insulin. After incubation for 1 h, cells were washed with PBS and incubated with lysis buffer (0.1 M potassium phosphate, 1% Triton X-100, pH 10) for 10 min with shaking. Subsequently, DMSO was added with shaking for 10 min. The fluorescence signal was measured with a microplate reader (FLUOstar OPTIMA, BMG LABTECH, Germany) at excitation and emission wavelengths of 466 nm and 540 nm, respectively.

### Western blot analysis

Cells and tissues were washed with ice-cold PBS, and lysed in RIPA buffer (50 mM NaCl, 10 mM Tris, 0.1% SDS, 1% Triton X-100, 0.1% sodium deoxycholate, 5 mM EDTA, 1 mM Na3VO4, pH 7.4). Total protein (40 µg) was separated by SDS-polyacrylamide gel electrophoresis and transferred to a nitrocellulose membrane (Whatman, Dassel, Germany). The membrane was blocked with 5% skim milk for 1 h and incubated with primary antibodies (diluted 1∶1000) overnight at 4°C. After washing with Tris-buffered saline containing 0.1% Tween-20, the membrane was incubated with HRP-conjugated secondary antibodies (diluted 1∶3000) for 1 h at room temperature. Antibody binding on the nitrocellulose membrane was detected with an enhanced chemiluminescence solution (Amersham Bioscience, Buckinghamshire, UK) and radiography. The intensity of each band was analyzed with a Lumino image analyzer (Model LAS-4000 Mini; Fujifilm, Tokyo, Japan) coupled with image analysis software (Multi Gauge Ver. 3.0; Fujifilm).

### Quantitative RT- PCR analysis

Total RNA was isolated from C_2_C_12_ myotubes and muscles and livers in mice using trizol (Invitrogen, Carlsbad, CA, USA), and cDNA was synthesized using a PrimeScript 1st strand cDNA synthesis kit (Takara Bio Inc., Shiga, Japan) according to the manufacturer's instructions. Real-time PCR was performed in triplicate using a FastStart SYBR Green Master (Roche Diagnostics, Mannheim, Germany) in an ABI Prism 7300 Sequence Detection System (Applied Biosystems, Foster City, CA, USA). The expression levels of the target genes relative to that of the endogenous reference gene actin were calculated using the delta cycle threshold method. The primer sequences are listed in Table S3.

### Statistical analysis

All experimental results were compared by one-way analysis of variance (ANOVA) using the Statistical Package for the Social Sciences (SPSS, ver. 11.0) program, and the data were expressed as means ± SE. Group means were considered to be significantly different at p<0.05 as determined by the technique of protective least significant difference when ANOVA indicated an overall significant treatment effect of p<0.05.

## Results

### BDSW suppresses fasting blood glucose levels and improves glucose intolerance in STZ-induced diabetic mice

**Figure 1:**
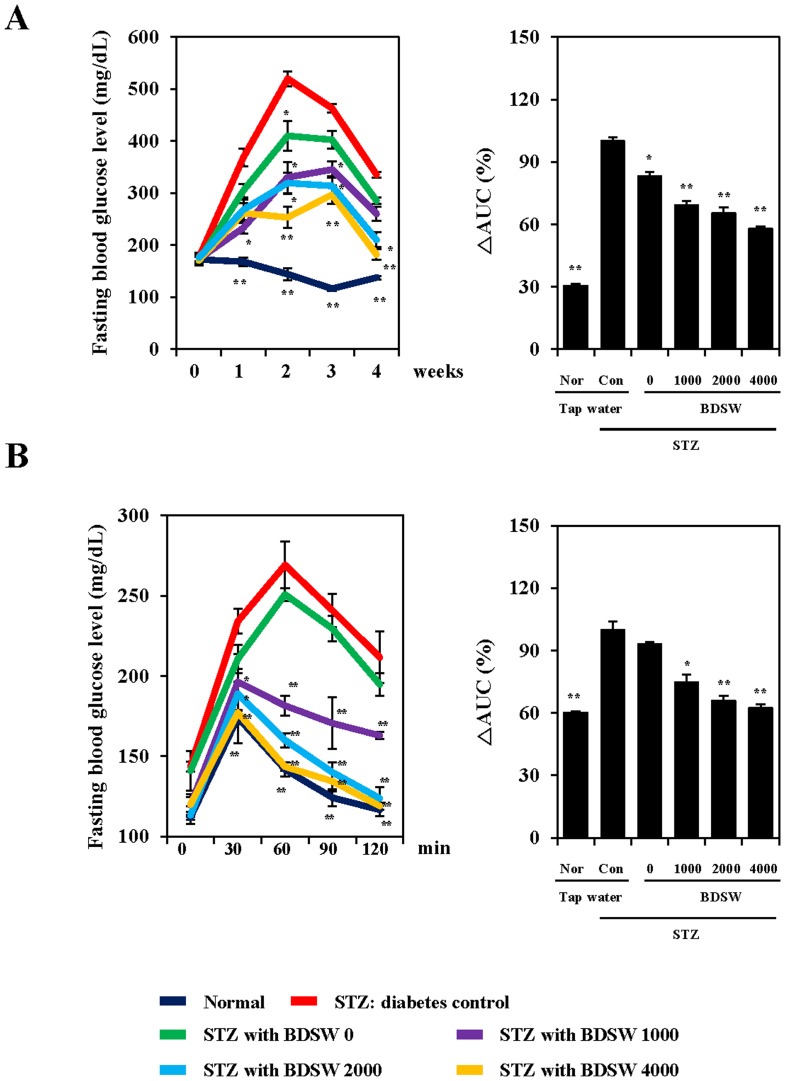
Effect of BDSW on fasting blood glucose levels (A) and glucose tolerance (B) in STZ-induced diabetic mice, for 4 weeks. Each color curve represents normal (dark blue), STZ diabetes control (Red), STZ with BDSW 0 (green), 1000 (purple), 2000 (deep Sky Blue), 4000 (yellow) group, respectively. Each value represents the mean ± SE (n = 8 per group). *P<0.05, **P<0.01: significant difference vs. STZ diabetic group. Nor, normal group; Con, STZ diabetic group; STZ, streptozotocin; BDSW, balanced deep-sea water.

To determine the effects of BDSW on STZ-induced diabetes development, we investigated fasting blood glucose levels and glucose tolerance. During 4 weeks of its drinking, BDSW attenuated the expected increase in the fasting blood glucose level in a dose-dependent manner (Fig 1A). Food intake did not differ between STZ and STZ with BDSW groups; however, there was a difference in BDSW intake between the BDSW groups. Body weight gain reduced in all STZ groups (Table S4). Triglycerides and total cholesterol levels in serum were increased by BDSW. In contrast, the triglycerides levels in liver were decreased by BDSW, and the total cholesterol levels were not different. In addition, we found that the serum insulin levels were increased in the STZ with BDSW mice (Table S4). To further evaluate the effect of BDSW on STZ-induced diabetes, we conducted OGTT after 4 weeks of BDSW drinking. The zero-time fasting blood glucose levels did not differ between groups. However, after glucose injection, all, except for BDSW (hardness 0 ppm), improved the impaired glucose tolerance induced by STZ (Fig 1B). These findings indicate that BDSW has strong anti-hyperglycemic potential in STZ-induced diabetic mice.

### BDSW increases adiponectin, leptin, and insulin levels in the plasma and improves pancreatic function of STZ-induced diabetic mice

**Figure 2:**
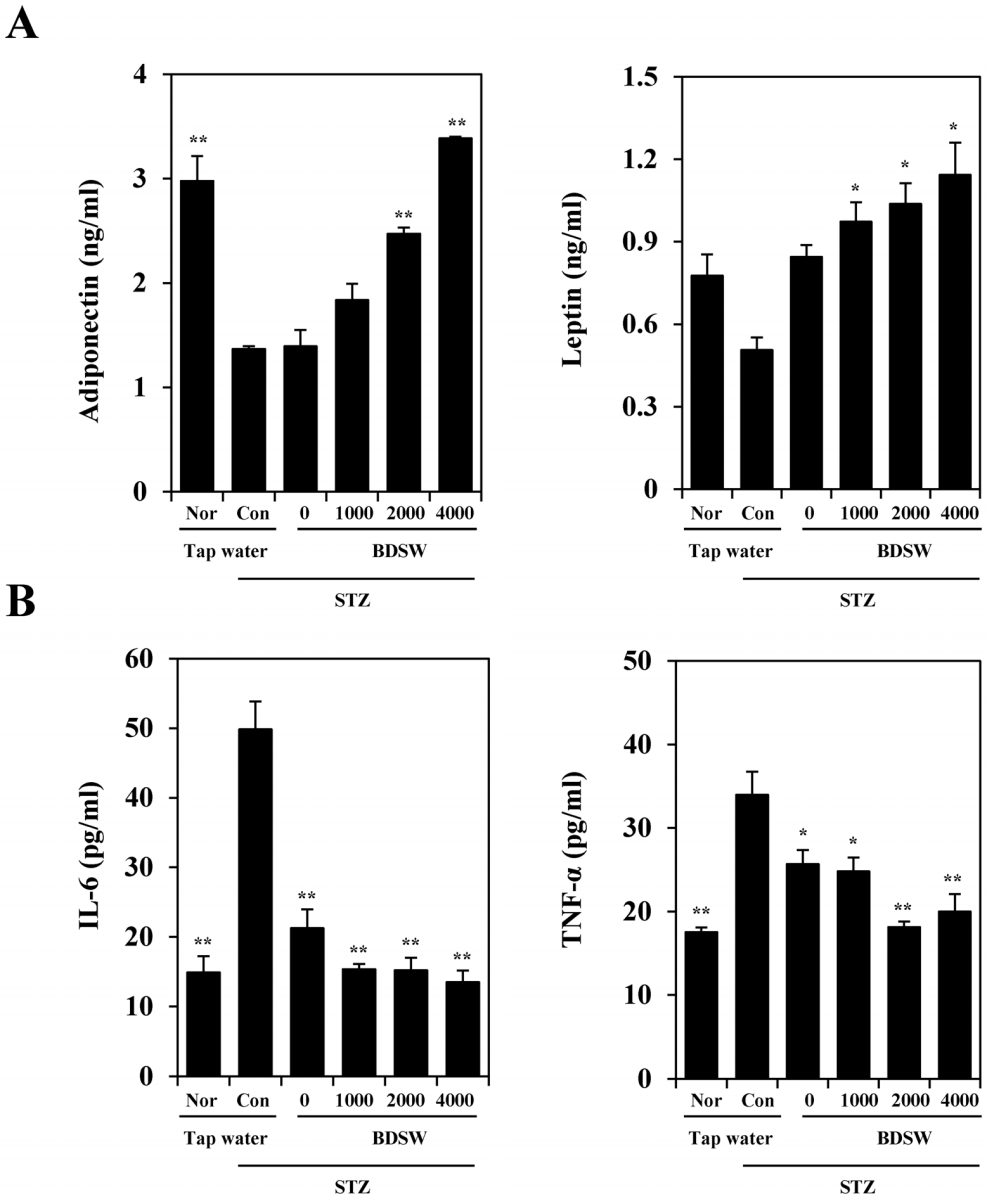
Effects of BDSW on adipokines (A) and cytokines (B) levels in plasma of STZ-induced diabetic mice, for 4 weeks. Each value represents the mean ± SE (n = 8 per group). *P<0.05, **P<0.01: significant difference vs. STZ diabetic group. Nor, normal group; Con, diabetic control group; STZ, streptozotocin; BDSW, balanced deep-sea water.

To further investigate the regulatory mechanism that link hyperglycemia and insulin resistance, we investigated the levels of the major insulin-sensitizing adipokines—adiponectin and leptin (Fig 2A)—as well as insulin (Table S4) in plasma from STZ-induced diabetic mice. BDSW increased the adiponectin and leptin levels and reduced the levels of the proinflammatory cytokines IL-6 and TNF-α (Fig. 2B). These findings suggest that BDSW regulates the physiological and molecular functions of adipokines in STZ-induced diabetic mice.

### BDSW improves the architecture of the pancreatic islets of Langerhans and insulin levels from beta cells of STZ-induced diabetic mice

**Figure 3:**
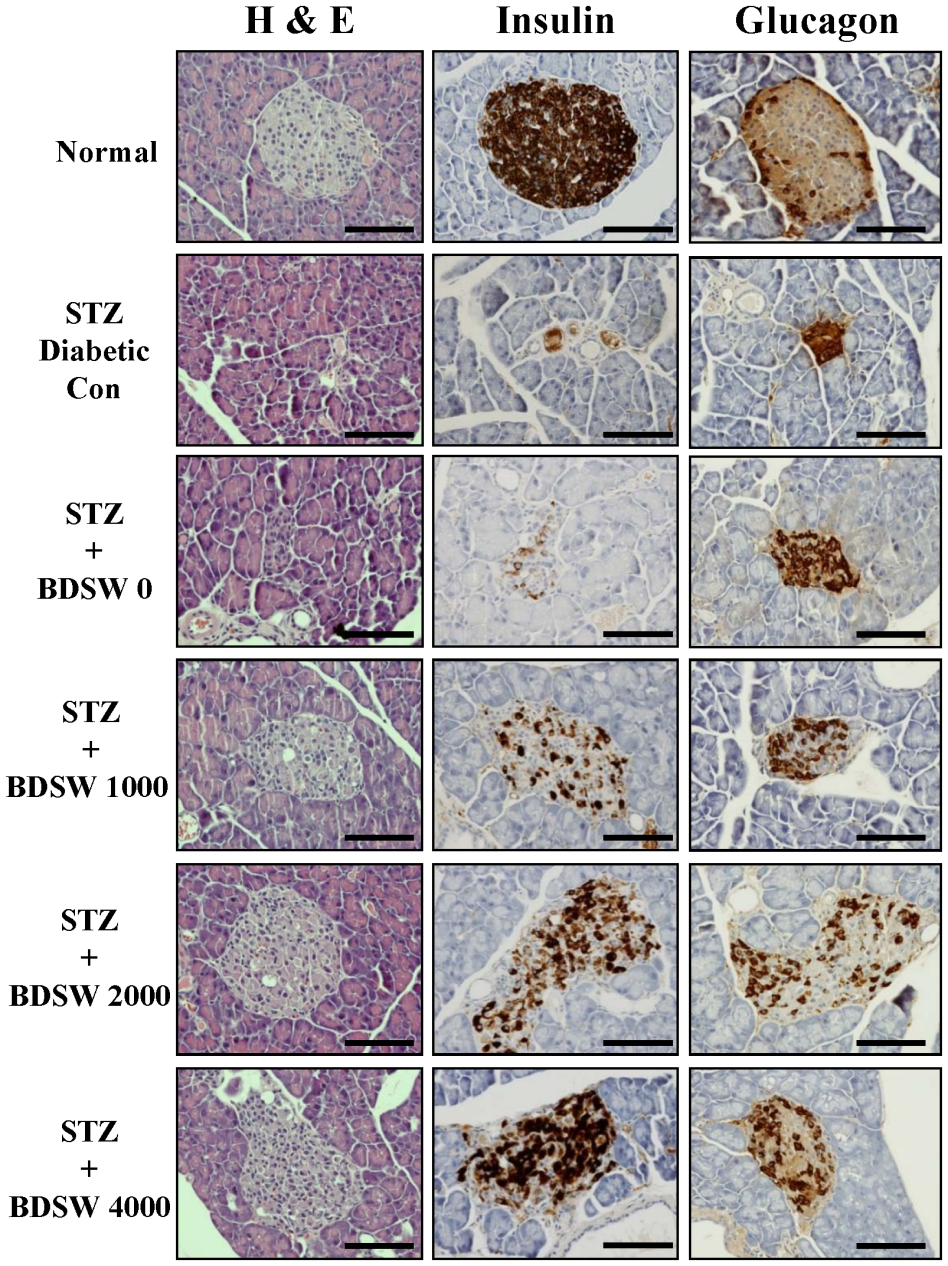
Effects of BDSW on pancreatic islet morphology and insulin and glucagon production in pancreas of STZ-induced diabetic mice, for 4 weeks. Representative hematoxylin and eosin, insulin, and glucagon staining of the islets of Langerhans are shown at ×400 magnification (n = 8 per group). Scale bar, 50 µm. STZ, streptozotocin; BDSW, balanced deep-sea water.

Histological analysis of the pancreas revealed that BDSW improves the architecture of pancreatic islets of Langerhans and enhances insulin secretion from β-cells of STZ-induced diabetic mice. The pancreatic β-cells were markedly and specifically lost in tissue sampled from the STZ control group. Morphological damage in pancreatic β-cells was prevented in the 1000–4000 BDSW group. In addition, insulin levels were found to be dependent on BDSW drinking (Fig 3 and Table S4). These findings suggest that the increase in insulin secretion in the BDSW group is likely associated with improved survival and function of the β-cells.

### BDSW regulates gene expression of glucose homeostasis proteins in the liver and muscle of STZ-induced diabetic mice

**Figure 4:**
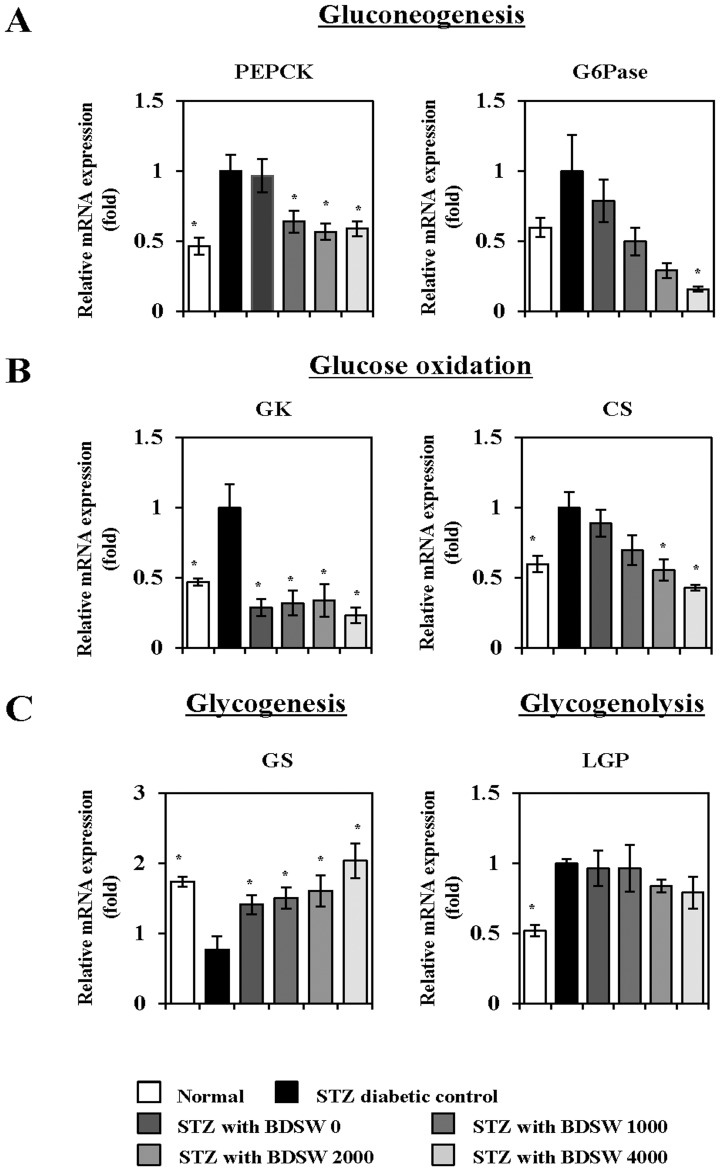
Effects of BDSW on the expression of genes involved in gluconeogenesis (A), glucose oxidation (B), and glycogen metabolism (C), in the liver of STZ-induced diabetic mice for 4 weeks. Each value represents the mean ± SE (n = 8 per group). *P<0.05, **P<0.01: significant difference vs. STZ diabetic group. STZ, streptozotocin; BDSW, balanced deep-sea water.

**Figure 5:**
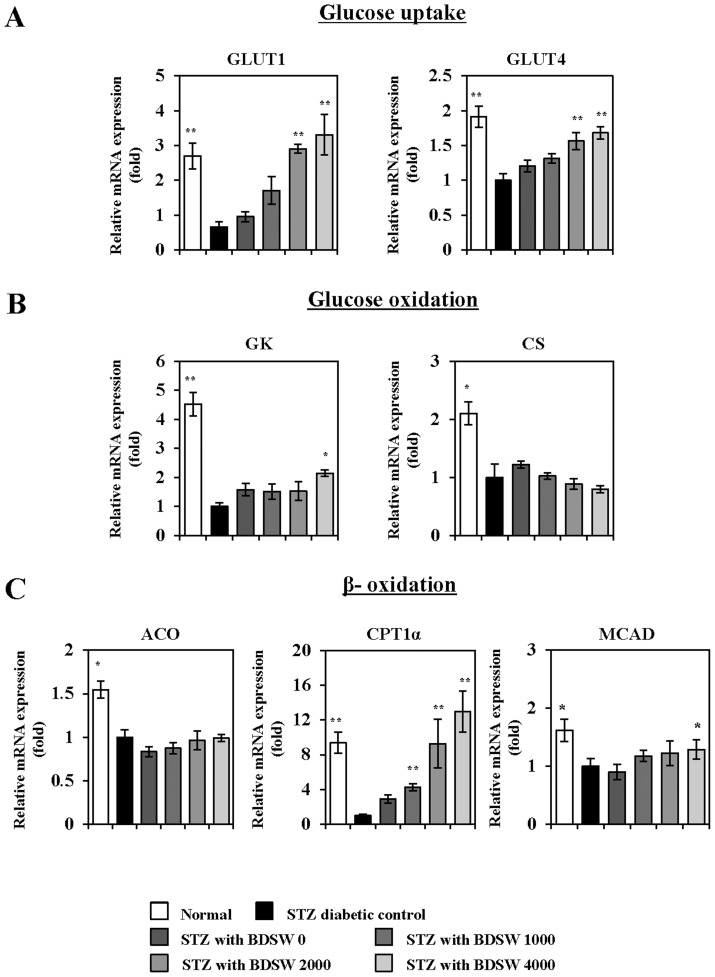
Effects of BDSW on the expression of genes involved in glucose uptake (A), glucose oxidation (B), and β-oxidation (C) in the muscles of STZ-induced diabetic mice for 4 weeks. Each value represents the mean ± SE (n = 8 per group). *P<0.05, **P<0.01: significant difference vs. STZ diabetic group. STZ, streptozotocin; BDSW, balanced deep-sea water.

Next, we investigated whether BDSW affects glucose homeostasis metabolism in the liver (specifically, gluconeogenesis, glycogenesis, glycogenolysis, and glucose oxidation) and glucose oxidation in skeletal muscle. Quantitative real-time PCR analysis revealed that BDSW downregulates the expression of phosphoenolpyruvate carboxykinase (PEPCK) and glucose 6-phosphatase (G6Pase), both of which are required for gluconeogenesis (Fig 4A); glucokinase (GK) and citrate synthase (CS), both of which are required for glucose oxidation (Fig 4B)); and liver glycogen phosphorylase (LGP), which is required for glycogenolysis (Fig 4C). Notably, glycogen synthase (GS) expression, which is critical for glycogenesis, was upregulated (Fig 4C). In contrast, in skeletal muscle, BDSW upregulated the expression of GLUT1 and GLUT4, which are required for glucose transport (Fig 5A); glucokinase and citrate synthase, which are required for glucose oxidation (Fig 5B); and acyl-CoA oxidase (ACO), carnitine palmitoyl transferase 1α (CPT1α), and mitochondrial medium-chain acyl-CoA dehydrogenase (MCAD), which are required for β-oxidation (Fig 5C). These findings suggest that BDSW regulates blood glucose levels by inhibiting glucose production and enhancing glucose uptake via regulation of gene expression.

### BDSW stimulates glucose uptake by C_2_C_12_ myotubes in a dose-dependent manner

**Figure 6:**
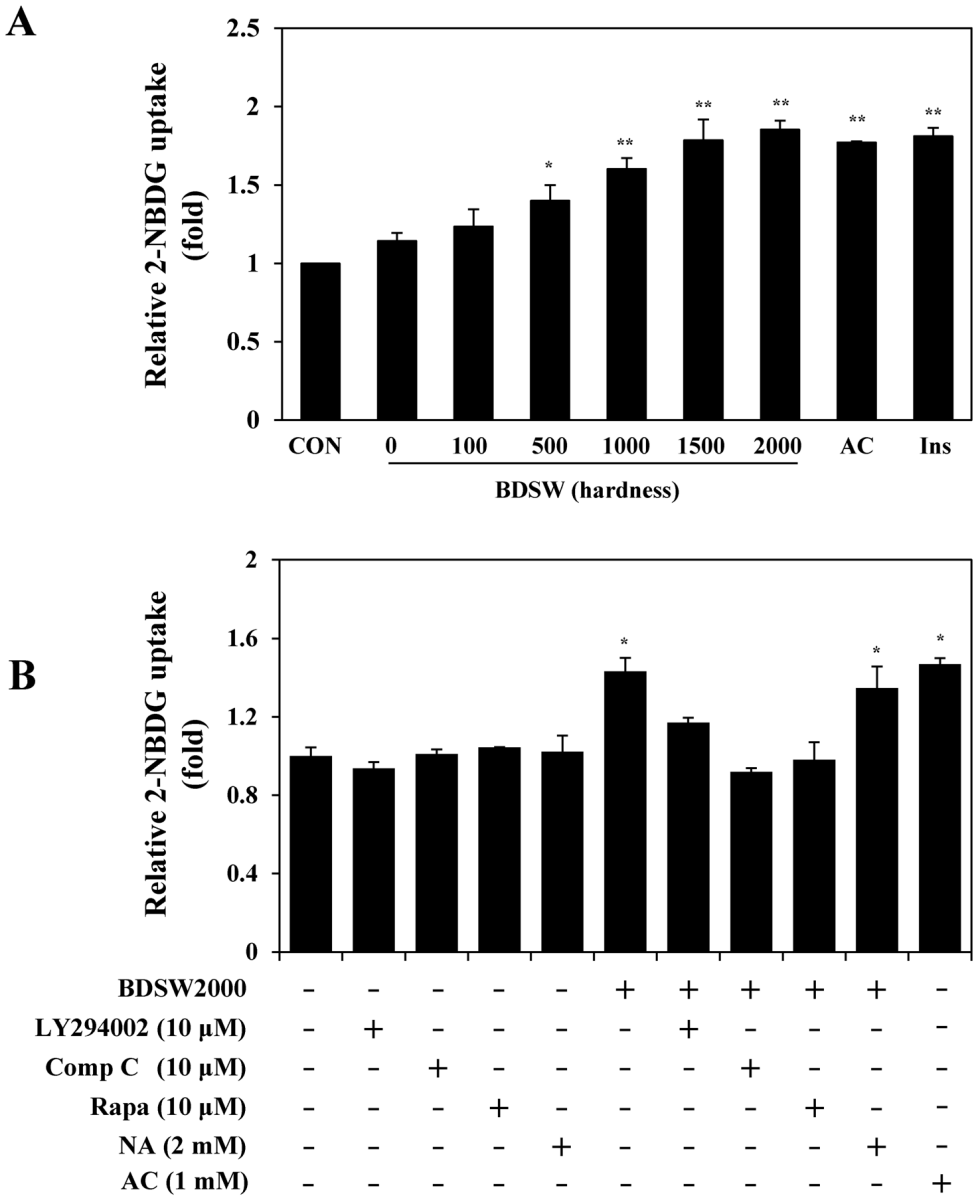
Effects of BDSW on 2-NBDG uptake in C_2_C_12_ myotubes. For the glucose-uptake assay at different levels of BDSW hardness (A), C_2_C_12_ myotubes were preincubated in DMEM with low glucose for 1 h. They were then incubated in DMEM containing BDSW at different levels of hardness, or 1 mM AICAR (AC), and 100 nM insulin (Ins) with 20 µM 2-NBDG for 1 h. For the glucose-uptake assay using several inhibitors (B), after preincubation in DMEM with low glucose in the presence or absence of 10 µM LY294002, 10 µM compound C (Comp C), 10 µM rapamycin (Rapa), 2 mM nicotinamide (NA), and 1 mM AICAR (AC) for 1 h, the cells were incubated in DMEM containing BDSW 2000 ppm hardness only, or with inhibitors, with 20 µM 2-NBDG for 1 h. After incubation was completed, cells were lysed, and the glucose uptake was measured using a fluorometer. Each value represents the mean ± SE (n = 8 per group). *P<0.05, **P<0.01: significant difference vs. CON group (A) or BDSW 2000 group (B). CON, non-treated control group; BDSW, balanced deep-sea water.

Owing to our in vivo findings, we aimed at determining whether BDSW directly stimulates glucose uptake; therefore, we performed a glucose-uptake assay to measure 2-NBDG uptake in C_2_C_12_ myotubes. BDSW significantly increased glucose uptake in a dose-dependent manner (Fig 6A). The increased glucose uptake induced by BDSW at a hardness of 1000–2000 ppm was similar to that induced by AICAR and insulin, both of which are agonists of glucose uptake in C_2_C_12_ myotubes. To investigate the regulatory mechanisms of BDSW-promoted glucose uptake, we next investigated the effects of several inhibitors on BDSW-promoted glucose uptake, namely, LY294002 (a potent inhibitor of PI3-K), compound C (an ATP-competitive inhibitor of AMPK), rapamycin (an inhibitor of mammalian target of rapamycin; mTOR), and nicotinamide (a Sirt1 inhibitor). BDSW-mediated promotion of glucose uptake by was inhibited by treatment with compound C and rapamycin, while LY294002 partially inhibited glucose uptake and nicotinamide did not affect glucose uptake (Fig 6B). These results suggest that the stimulatory effect of BDSW on glucose uptake is dependent on the PI3-K, AMPK and mTOR pathways.

### BDSW specifically stimulates the phosphorylation of IRS-1, LKB1, AMPK, and mTOR, and also improves the impaired phosphorylation of these molecules in the muscles of mice with STZ-induced non-obese diabetes

**Figure 7:**
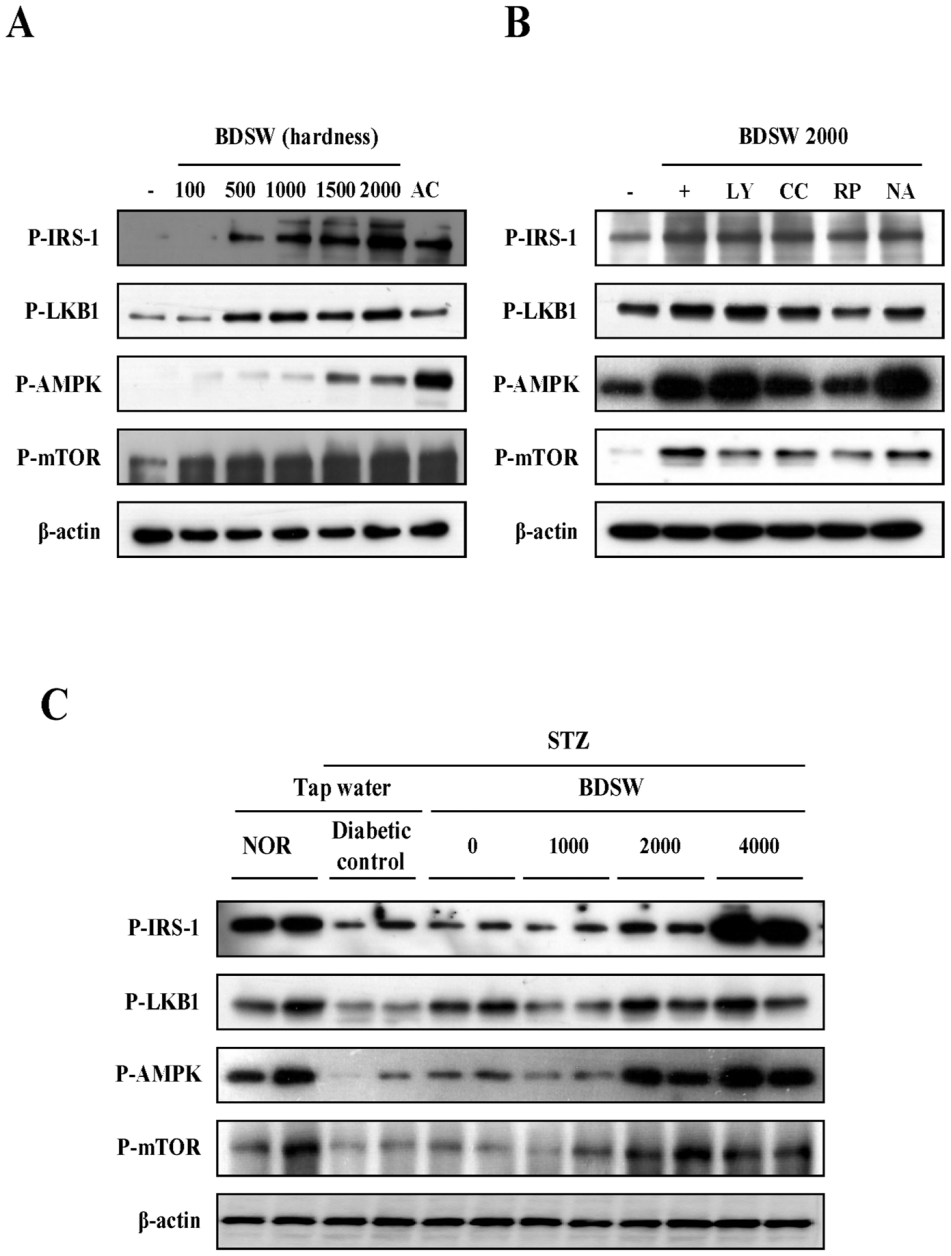
Effects of BDSW on the phosphorylation of IRS-1, LKB, AMPK, and mTOR in C_2_C_12_ myotubes (A, B) and the muscles (C) of STZ-induced diabetic mice. C_2_C_12_ myotubes were preincubated in DMEM with low glucose for 1 h. They were then incubated in DMEM containing BDSW at different levels of hardness and 1 mM AICAR (AC) for 1 h. For inhibitors assay, after preincubation in DMEM with low glucose in the presence or absence of 10 µM LY294002, 10 µM compound C (CC), 10 µM rapamycin (RP), and 2 mM nicotinamide (NA) for 1 h, the cells were then incubated in DMEM containing BDSW of 2000 ppm hardness with or without inhibitors for 1 h. Subsequently, lysates (20 µg) of C_2_C_12_ myotubes were subjected to SDS-PAGE and western blotting analyses using anti-phospho IRS-1, anti-phospho LKB1, anti-phospho AMPK, anti-phospho mTOR, anti-AMPK, and anti-β-actin antibodies. NOR, normal group; STZ, streptozotocin; BDSW, balanced deep-sea water.

To accurately determine the regulatory mechanism by which BDSW stimulates glucose uptake, we investigated the phosphorylation of IRS-1, LKB1, AMPK, and mTOR, which are major signal molecules related to glucose uptake, in C_2_C_12_ myotubes. BDSW specifically stimulated the phosphorylation of IRS-1, LKB1, AMPK, and mTOR in a dose-dependent manner (Fig 7A). BDSW-mediated LKB1 and AMPK phosphorylation was inhibited by pretreatment with compound C and rapamycin. However, the phosphorylation of IRS-1 was not affected by the inhibitors except for rapamycin (Fig 7B). Moreover, BDSW at hardness of 2000–4000 ppm increased the phosphorylation of IRS-1, LKB1, AMPK, and mTOR in muscles of STZ-induced diabetic mice (Fig 7C). Therefore, these results suggest that BDSW increases glucose uptake through the IRS-1, LKB1-AMPK, and mTOR pathways.

## Discussion

In the present study, we demonstrated that BDSW inhibits hyperglycemia, improves glucose intolerance and pancreatic function, and increases glucose uptake through the phosphorylation of signal molecules related to glucose uptake such as IRS-1, LKB1, AMPK, and mTOR in the skeletal muscle of STZ-induced diabetic mice.

The role of DSW in glucose metabolism was proposed in previous reports on type 2 diabetes in *ob/ob* mice [15] and high-fat diet–induced obese diabetic mice [12]. However, the DSW concentration required to treat or prevent metabolic disease as well as the detailed regulatory mechanisms involved remain unclear. Moreover, there is no scientific evidence regarding the effect of DSW on non-obese diabetes or type 1 diabetes. Therefore, in this study, we used BDSW of varying hardness, ranging from 1000 to 4000 ppm, and examined the effects of BDSW treatment on STZ-induced type 1 diabetic mice.

BDSW has high contents of unique minerals such as Mg, Ca, and K. Among many kinds of minerals, Mg plays a major role in disease prevention and overall health. Low Mg levels are associated with several chronic diseases including migraine headaches, Alzheimer's disease, stroke, hypertension, cardiovascular disease, and type 2 diabetes mellitus [16]. Mg plays pivotal roles in glucose homeostasis and insulin sensitivity [17]. The intake of a Mg-deficient high-fat diet led to alterations in the insulin-signaling pathway and consequently increased insulin resistance [18]. On the other hand, Mg supplementation delays the development of diabetes in Otsuka Long-Evans Tokushima Fatty (OLETF) rats [19]. Ca supplementation protects against stress-induced osmotic fragility of red blood cells in patients with type 2 diabetes mellitus [20]. Higher K intake is significantly associated with a lower metabolic syndrome prevalence in women [21]. Therefore, the consistent supply of minerals from BDSW drinking may be beneficial in the prevention of diabetes. BDSW also has diverse trace minerals such as Se, Zn, and V. Trace minerals are a group of minerals required by the body in very small quantities. Although its exact physiological actions are not yet known, vanadium is believed to be an important trace element, since its deficiency results in a variety of problems such as reproductive problems and skeletal abnormalities [22]. In particular, vanadium likely plays a significant role in thyroid, iron, glucose, and lipid metabolism [23]. The effects of selenium [24] and zinc [25] on diabetic cardiovascular complications are similar to that of vanadium. Therefore, in future studies, we also plan to examine, in detail, the use of trace minerals derived from DSW mineral extracts in the treatment of obese and non-obese diabetes.

The dysregulation of adipokines has been implicated in obesity, diabetes, and cardiovascular disease [26]. Adiponectin and leptin are known anti-obesity and anti-diabetic adipokines. Adiponectin is an insulin-sensitizing hormone that exhibits direct anti-diabetic, anti-atherogenic, and anti-inflammatory potential. Adiponectin also modulates a number of metabolic processes, including glucose and fatty acid metabolism [27]. Leptin represses food intake and promotes energy expenditure [28]. Our findings revealed that BDSW increased adiponectin, leptin, and insulin levels in a dose-dependent manner. Other studies indicate that leptin functions in adiposity signaling, since its levels in the blood correlate with body fat. Although several studies suggest that leptin is important for adiposity signaling, it is likely not the only molecule involved in adiposity signaling, and therefore, at least one additional signal molecule or protein must be involved in adiposity signaling: A logical candidate is the pancreatic hormone insulin. Plasma insulin levels directly correlate with adiposity and visceral fat levels [29]. Leptin and insulin together provide information to the brain about the size of the fat mass, its distribution, and important changes in metabolism. Therefore, the increase in these adipokines level caused by BDSW seems to due to normalization of body adiposity and pancreatic function, including insulin production, in beta cells. However, a more detailed investigation of the regulatory mechanism of BDSW-induced adiponectin, leptin, and insulin production is still needed. A recent study showed that inflammatory responses in adipose tissue induce peripheral tissue insulin resistance [30]. In obese humans [31], the expression of pro-inflammatory adipokines is enhanced to induce insulin resistance. These pro-inflammatory cytokine levels were also reduced with BDSW treatment. Together, these findings suggest that BDSW improves insulin resistance by regulating the physiological and molecular functions of cytokines in STZ-induced diabetic mice.

On the other hand, histological assay of the pancreas revealed that BDSW improves the architecture of the pancreatic islets of Langerhans and insulin production from beta cells in the BDSW group. Insulin levels were markedly increased in mice fed with BDSW (hardness, 4000 ppm). The increase of insulin levels in the BDSW group may be associated with the improvement of beta cell survival. However, further detailed studies are needed to elucidate the regulatory mechanism of BDSW-induced insulin production.

Several studies have shown that DSW inhibits lipid metabolism, including lipogenesis and cholesterol synthesis. In particular, drinking of DSW with high levels of Mg reduced serum lipids [32] and alleviated hepatic lipid accumulation and oxidation induced by high-fat diet [11] [33]. We also found that BDSW suppressed circulating triglycerides levels, except total cholesterol level, in the liver of STZ induced diabetic mice. In contrast, BDSW enhanced expression of genes related to processes of glycogen production including glycogenesis, and inhibited the expression of genes related to processes of glucose output, such as glucogenesis as well as glucose oxidation. In skeletal muscle, which aids in the maintenance of blood glucose concentration, BDSW increased gene expression of glucose transporters, glucose oxidation, and β-oxidation. These results suggest that BDSW may improve insulin resistance through the regulation of glucose and lipid metabolism.

Insulin-stimulated glucose uptake by adipocytes and skeletal muscle play an important role in the maintenance of whole-body glucose homeostasis [34]. BDSW increased the activity of signal molecules related to glucose uptake, such as AMPK, in C_2_C_12_ myotubes. AMPK has emerged as a major cellular energy sensor and master regulator of metabolic homeostasis in glucose and lipid metabolism. It is activated by a decrease in the cell's energy level, which is reflected by an increased AMP/ATP ratio. AMPK has been shown to mediate the metabolic effects of hormones such as leptin, ghrelin, adiponectin, glucocorticoids and insulin as well as cannabinoids. Generally, activated AMPK stimulates catabolic pathways such as glycolysis, fatty acid oxidation and mitochondrial biogenesis and inhibits anabolic pathways such as gluconeogenesis, glycogen, fatty acid and protein synthesis, and has a direct appetite-regulating effect in the hypothalamus [35]. AMPK also is considered an attractive therapeutic target for metabolic diseases including obesity and diabetes [36]. Our findings also showed that BDSW-induced glucose uptake was independent of insulin and dependent on the AMPK and mTOR pathways. Moreover, BDSW activated AMPK both in vitro and in vivo. These results suggest that BDSW may improve insulin resistance through the regulation of glucose and lipid metabolism.

In conclusion, our findings indicate that inhibition of hyperglycemia and improvement of glucose intolerance by BDSW may be mediated, at least in part, by the downregulation of genes related to gluconeogenesis, glycogenolysis, and glucose oxidation processes in the liver as well as by the upregulation of genes related to glucose uptake in skeletal muscle. Additionally, BDSW restored LKB1, AMPK, and mTOR activation both in vitro and in vivo. The findings of present study are concordant with those of our previous study on the underlying cellular or molecular mechanism by which BDSW influences high-fat-diet–induced obese diabetic mice and 3T3-L1 adipocytes. Therefore, BDSW could be a potential anti-hyperglycemic and lipid-lowering therapeutic agent or medicinal health food for the prevention or treatment of type 1 and type 2 diabetes. In a future study, we plan to apply for clinical trial using minerals extracted from deep-sea water.

## Supporting Information

Table S1 **Mineral content of original DSW and balanced DSW used in this study (Ex. Hardness 4680).**
(TIFF)

Table S2 **The standard ingredients of CRF-1 used in this study (per 100 g).**
(TIFF)

Table S3 **Primers for quantitative real-time polymerase chain reaction analysis.**
(TIFF)

Table S4 **Effects of BDSW on body weight, food intake, serum/liver triglycerides and total cholesterol levels, and serum insulin levels in STZ-induced diabetic mice.** Body weight, food intake, tap water and BDSW intake were measured once every three days for 4 weeks. The levels of triglycerides and total cholesterol in serum and liver, and insulin in serum were measured at 4 weeks of BDSW drinking. Each value represents the mean ± SE (n = 8 per group). *P<0.05, **P<0.01: significant difference vs. STZ control group. NOR, normal group; BDSW, balanced deep-sea water.
(TIFF)
